# A further case of *AFG2B*‐related neurodevelopmental disorder with hearing loss and microcephaly allows further clarification of pathogenicity of the variant c.1313T>C, p.(Leu438Pro)

**DOI:** 10.1002/mgg3.2310

**Published:** 2023-10-30

**Authors:** Sarah Grosch, Martin Kehrer, Olaf Riess, Andrea Bevot, Tobias B. Haack

**Affiliations:** ^1^ Institute of Medical Genetics and Applied Genomics University of Tübingen Tübingen Germany; ^2^ Centre for Rare Diseases University of Tübingen Tübingen Germany; ^3^ Department of Neuropediatrics, Developmental Neurology and Social Pediatrics University of Tübingen Tübingen Germany

**Keywords:** AFG2B, neurodevelopmental disorder, trio exome sequencing

## Abstract

**Background:**

Bi‐allelic variants in *AFG2B* (previously known as *SPATA5L1*) have recently been associated with a neurodevelopmental disorder with hearing loss and spasticity, as well as isolated hearing loss. We report on a 6 1/2‐year‐old girl with a history of global developmental delay, subsequent intellectual disability without relevant language acquisition, sensorineural hearing loss, muscular hypotonia and microcephaly.

**Methods:**

We performed trio exome sequencing on the patient and her parents.

**Results:**

Trio exome sequencing revealed likely pathogenic compound heterozygous missense variants in *AFG2B* [c.527G>T, p.(Gly176Val) and c.1313T>C, p.(Leu438Pro)] in the patient.

**Conclusion:**

Of note, the change c.1313T>C, p.(Leu438Pro) has been observed in a previously published patient as part of a complex disease allele along with a second homozygous missense change, so the exact contribution of the two alterations to this patient's disease had initially remained unclear. Our results support the pathogenic relevance of the c.1313T>C, p.(Leu438Pro) allele while providing detailed insights into the disease manifestation of a further patient.

## Graphical abstract

In a 6 1/2‐year‐old girl with a neurodevelopmental disorder, we identified compound heterozygous variants in *AFG2B* [c.527G>T, p.(Gly176Val) and c.1313T>C, p.(Leu438Pro)] by using trio exome sequencing. Our result support the pathogenic relevance of the variant [c.1313T>C, p.(Leu438Pro)], which had initially remained unclear in a previously described patient.

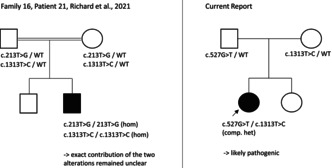

## 1 INTRODUCTION

*AFG2 AAA ATPase homolog B* (*AFG2B*, previously known as *SPATA5L1*), a paralog of *AFG2A*, belongs to the AAA+ ATPases protein superfamily (Liu et al., 2000; Richard et al., 2021) and, together with *C1orf109*, *AFG2A* and *CINP*, plays an essential role in human ribosomal pre‐60S maturation in the cytoplasm (Ni et al., 2022). Recently, bi‐allelic variants in *AFG2B* have been associated with neurodevelopmental disorder with hearing loss and spasticity (OMIM #619616), as well as with isolated hearing loss (OMIM #619615) (Richard et al., 2021). In total, 28 bi‐allelic variants in *AFG2B* in 47 individuals with hearing loss have been reported so far. In total, 25 individuals showed a neurodevelopmental disorder in addition to hearing loss, characterised by global developmental delay, spastic‐dystonic cerebral palsy, focal or generalised epilepsy and microcephaly (Richard et al., 2021). Among them, a patient carrying two rare homozygous variants, c.213T>G, p.(Phe71Leu) and c.1313T>C, p.(Leu438Pro) [Patient 21 (Richard et al., 2021)]. At that time, it had remained unclear which of the two homozygous variants or whether their combination was causative for his disease. We report on a 6 1/2‐year‐old girl with a neurodevelopmental disorder carrying compound heterozygous missense variants in *AFG2B* [c.527G>T, p.(Gly176Val) and c.1313T>C, p.(Leu438Pro)]. While providing a detailed clinical report on the girl's neurodevelopmental disease presentation, our results also argue for the clinical relevance of the c.1313T>C, p.(Leu438Pro) variant.

## 2 MATERIALS AND METHODS

### 2.1 Ethics statement

The study was approved by the Institutional Review Board of the Ethics Committee of the University Hospital of Tübingen under the study number 116/2015BO2 and written informed consent for genetic testing and publication was obtained from the patient's parents.

### 2.2 Genetic analysis

Diagnostic trio exome sequencing (ES) was performed on DNA extracted from peripheral blood from the proband and her parents. Coding genomic regions were enriched using a SureSelect XT Human All Exon Kit V.7 (Agilent Technologies, Santa Clara, California, USA) for subsequent sequencing as 2 × 100 bp paired‐end reads on a NovaSeq6000 system (Illumina, San Diego, California, USA). Generated sequences were analysed using the megSAP pipeline (https://github.com/imgag/megSAP). Clinical variant prioritisation included different filtering steps, e.g. minor allele frequency (MAF) ≤1% in gnomAD (https://gnomad.broadinstitute.org) and in an in‐house database. In addition, a phenotype‐based filtering was performed. DNA variants were interpreted in the context of the parents' variants and classified following the guidelines of the American College of Medical Genetics and Genomics (ACMG) (Richards et al., 2015). Sanger sequencing was performed to confirm identified *AFG2B* variants. Primer sequences and PCR conditions are available upon request.

## 3 RESULTS

### 3.1 Clinical summary

We report on a 6 1/2‐year‐old girl with severe sensorineural hearing loss, global developmental delay and intellectual disability without relevant language acquisition and microcephaly. She was the first child of nonconsanguineous, healthy parents, whose families originate from Germany and Scandinavia. A 3‐year‐old sister is healthy. Aside from presbycusis, family history was negative. Pregnancy was normal except from gestational diabetes. The girl was born at a gestational age of 36 4/7 weeks. Body measurements were normal; birth weight was 3220 g (78th percentile, 0.77z), birth length was 50 cm (65th percentile, 0.38z) and head circumference was 33.5 cm (46th percentile, −0.11z).

Postnatal hearing screening revealed pathological findings, and BERA at the age of 6 months was abnormal. The provision of hearing aids was started at the age of 9 months, leading to an acoustical response in the sense of normal hearing. Unilateral cochlear implantation was planned shortly after the presentation in our outpatient clinic. Postnatally, hydronephrosis was diagnosed, which spontaneously resolved. There were no organic malformations. She did not show any distinctive dysmorphic features, but a thick vermilion of the lower lip and small nevi flammei in the sacral area and on the left heel were noted. She showed muscular hypotonia and pedes valgi. There were no pyramidal signs or abnormal tendon reflexes, and cerebral MRI at the age of 2 years was normal. While length and weight followed the 90th percentiles, she developed microcephaly slightly below the 3rd percentile. Motor milestones were achieved on time, but slight clumsiness was noted. Language acquisition was not achieved before the age of 5 years. At the age of 5 3/12 years, she used 5 to 10 single words. Listening comprehension was adequate in both languages of the bilingual education. Socio‐emotional and cognitive development was delayed. She liked to arrange or sort simple, constructive play material or to play with modelling clay. She was friendly and interested in the activities of other children. There was a need for complex special needs education (accompanying person for the kindergarten, speech therapy and early hearing therapy). The family's resource‐intense psychosocial care included a disability card, special needs education and several interprofessional appointments and diagnostic procedures. Conventional chromosomal analyses and a panel‐based screening for genes associated with hearing loss (in 2018) revealed normal results. The following table shows the phenotypic characteristics of the patient in comparison to the characteristics of the previously described patients (Table 1).

**TABLE 1:** Comparison of phenotypic characteristics of our patient with previously described patients.

	Current report	Richard et al. (2021)
Intellectual disability	+	25/25
Hearing impairment	+	25/25
Spasticity	–	17/25
Dystonia	–	15/25
Hypotonia	+	17/25
Epilepsy	–	16/25
Microcephaly	+	13/25
Facial dysmorphism	(−)	09/25
	Thick vermilion of the lower lip and small nevi flammei in the sacral area and on the left heel	
Abnormal MRI	–	17/25
Visual impairment	–	15/25

*Note*: +, clinical feature detected; −, clinical feature not observed, Richard et al. (2021) reported 25 patients with neurodevelopmental disorder and hearing loss. The table shows how many of the patients present the phenotypic characteristics.

### 3.2 Molecular genetic results

**FIGURE 1:**
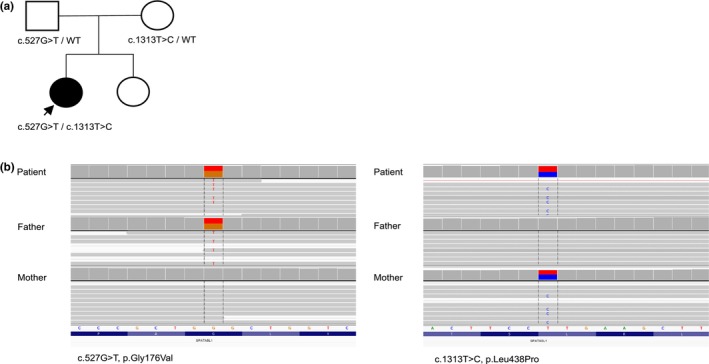
(a) The pedigree of our patient. Circles represent females. Squares represent males. The blank symbols represent the unaffected family members, and the black symbol represents the affected individual. WT Wildtype. (b) Visualisation of the compound heterozygous variants in the *AFG2B*‐Gene (previously known as *SPATA5L1*) in the Integrative Genome Viewer (IGV). The variants were identified through whole exome sequencing. The variant c.527G>T, p.Gly176Val was inherited from the patient's father. The variant c.1313T>C, p.Leu438Pro was inherited from the patient's mother. Variant coverage: c.527G>T, p.Gly176Val: patient: total read count: 415, G: 234, T: 181; father: total read count: 400, G: 208, T:192; mother: total read count: 458, G: 455, T: 2; c.1313T>C, p.Leu438Pro: patient: total read count: 109, T:57, C: 51; father: total read count: 120, T: 120; C: 0; mother: total read count: 119, T: 56, C: 63.

In the patient, we identified the compound heterozygous missense variants in *AFG2B* (NM_024063.3), c.527G>T, p.(Gly176Val) and c.1313T>C, p.(Leu438Pro), with the change c.527G>T, p.(Gly176Val) being located on the paternal and the change c.1313T>C, p.(Leu438Pro) on the maternal allele (Figure 1). Both variants were confirmed by Sanger sequencing. Details of the two identified variants are provided in the supplement (Table S1).

## 4 DISCUSSION AND CONCLUSION

The phenotypic features of the patient with *AFG2B* variants reported in this study overlap with the features of previously described patients with *AFG2B*‐related neurodevelopmental disease with hearing loss and neurological phenotypes.

The variant c.527G>T, p.(Gly176Val) has been reported in compound heterozygous form in three patients with neurodevelopmental disorder and hearing loss and spasticity (Patient 18, 19, 20 [Richard et al., 2021]). Moreover, this variant has been reported several times in compound heterozygous form in patients with isolated hearing loss (Richard et al., 2021). According to the ACMG‐Guidelines this variant is classified as pathogenic (PS1, PM1, PM3, PP4, PP5) (Richards et al., 2015).

The variant c.1313T>C, p.(Leu438Pro) has been reported in a single patient with a neurodevelopmental disorder, hearing loss and spasticity in homozygous form (Patient 21 [Richard et al., 2021]). This patient also carried a second rare homozygous variant in *AFG2B*, c.213T>G, p.(Phe71Leu). Hitherto, it was unclear which of the two variants alone is disease‐causing or whether their combination is required for functionally relevant impairment of *AFG2B*.

Based on the detection of the variant c.1313T>C, p.(Leu438Pro) in our patient in compound heterozygosity with a known pathogenic variant, we can clarify not only the cause of our patient's disease but also the probable cause of disease of the patient described previously. However, we cannot completely exclude the possibility that the variant c.1313T>C, p.(Leu438Pro) may be a hypomorphic variant, which does not lead to the manifestation of the full clinical phenotype in homozygous form, but only in combination with a second pathogenic variant in *AFG2B*. According to the ACMG‐Guidelines, the variant c.1313T>C, p.(Leu438Pro) is classified as likely pathogenic (PM2, PM3, PP3, PP4, PP5) and the variant c.213T>G, p.(Phe71Leu) is classified as a variant of uncertain significance (PMS2, BP2) (Richards et al., 2015). Speculatively, in the published individual 21, additive functional effects of the two homozygous alterations may contribute to the more severe clinical picture with disease onset in the first weeks of life, severe developmental delay and presumed central blindness, severe bilateral hearing loss and changes in muscle tone from hypotonia to dystonia to severe spasticity with contracture at 3.5 years of age.

In conclusion, our case report has contributed to further clarification of the pathogenicity of the variant c.1313T>C, p.(Leu438Pro). This allows a faster diagnosis of other patients carrying the same variant.

## AUTHOR CONTRIBUTIONS

*Study conception and design*: Tobias Haack, Martin Kehrer and Sarah Grosch. *Data collection*: Andrea Bevot and Martin Kehrer. *Analysis and interpretation of results*: Martin Kehrer, Tobias Haack, Olaf Rieß and Sarah Grosch. *Draft and/or revised manuscript preparation*: Martin Kehrer, Tobias Haack and Sarah Grosch. All authors reviewed the results and approved the final version of the manuscript.

## CONFLICT OF INTEREST STATEMENT

The authors declare no competing interests.

## Supporting information


Table S1:

## Data Availability

The data that support the findings of this study are available from the corresponding author upon reasonable request.

## References

[mgg32310-bib-0001] Liu, Y. , Black, J. , Kisiel, N. , & Kulesz‐Martin, M. F. (2000). Spaf, a new AAA‐protein specific to early spermatogenesis and malignant conversion. Oncogene, 19(12), 1579–1588. 10.1038/sj.onc.1203442 10734318

[mgg32310-bib-0002] Ni, C. , Schmitz, D. A. , Lee, J. , Pawłowski, K. , Wu, J. , & Buszczak, M. (2022). Labeling of heterochronic ribosomes reveals C1ORF109 and SPATA5 control a late step in human ribosome assembly. Cell Reports, 38(13), 110597. 10.1016/j.celrep.2022.110597 35354024 PMC9004343

[mgg32310-bib-0003] Richard, E. M. , Bakhtiari, S. , Marsh, A. P. L. , Kaiyrzhanov, R. , Wagner, M. , Shetty, S. , Pagnozzi, A. , Nordlie, S. M. , Guida, B. S. , Cornejo, P. , Magee, H. , Liu, J. , Norton, B. Y. , Webster, R. I. , Worgan, L. , Hakonarson, H. , Li, J. , Guo, Y. , Jain, M. , … Kruer, M. C. (2021). Bi‐allelic variants in SPATA5L1 lead to intellectual disability, spastic‐dystonic cerebral palsy, epilepsy, and hearing loss. American Journal of Human Genetics, 108(10), 2006–2016. 10.1016/j.ajhg.2021.08.003 34626583 PMC8546233

[mgg32310-bib-0004] Richards, S. , Aziz, N. , Bale, S. , Bick, D. , Das, S. , Gastier‐Foster, J. , Grody, W. W. , Hegde, M. , Lyon, E. , Spector, E. , Voelkerding, K. , & Rehm, H. L. (2015). Standards and guidelines for the interpretation of sequence variants: A joint consensus recommendation of the American College of Medical Genetics and Genomics and the Association for Molecular Pathology. Genetics in Medicine, 17(5), 405–424. 10.1038/gim.2015.30 25741868 PMC4544753

