# Supplementary material for: A further case of AFG2B‐related neurodevelopmental disorder with hearing loss and microcephaly allows further clarification of pathogenicity of the variant c.1313T>C, p.(Leu438Pro)
Source: Mol Genet Genomic Med. 2023 Oct 30;12(1):e2310. doi: 10.1002/mgg3.2310 (PMC10767672; doi:10.1002/mgg3.2310)
Supplement: Supplementary file 1 — Table S1: [file MGG3-12-e2310-s001.docx]

**Table S1**. Details of the identified variants in *AFG2B*

| **Position (hg 38)** | **Gene** | **Variant type** | **Variant** | **phyloP** | **CADD** | **gnomAD Frequency v3.1.2** | **Classification** |
| --- | --- | --- | --- | --- | --- | --- | --- |
|  |  |  | **(according to the transcript NM_024063.3)** |  |  |  | **(according to the ACMG-Guidelines)** |
| chr15:45402956 | AFG2B (SPATA5L1) | Missense | c.527G>T, p.Gly176Val | 37.430 | 19.27 | 148 het/152216 | Pathogenic |
|  |  |  |  |  |  |  | PS1, PM1, PM3, PP4, PP5 |
| chr15:45410409 | AFG2B (SPATA5L1) | Missense | c.1313T>C, p.Leu438Pro | 18.280 | 22.60 | 1 het/152236 | Likely pathogenic |
|  |  |  |  |  |  |  | PM2, PM3, PP3, PP4, PP5 |

hg38 Human genome build 38

phyloP (phylogenetic p-values)

CADD (Combined Annotation Dependent Depletion, <https://cadd.gs.washington.edu/>

gnomAD (Genome Aggregation Database), <https://gnomad.broadinstitute.org/>

ACMG-Guidelines (American College of Medical Genetics and Genomics)
